# Quantitative evaluation of multiple treatment regimens for treatment-resistant depression

**DOI:** 10.1093/ijnp/pyaf007

**Published:** 2025-01-25

**Authors:** Yulin Feng, Yinghua Lv, Juan Yang, Ling Xu, Junchao Chen, Jihan Huang, Jiyuan Ren, Qingshan Zheng, Lujin Li

**Affiliations:** Center for Drug Clinical Research, Shanghai University of Traditional Chinese Medicine, Shanghai, China; Center for Drug Clinical Research, Shanghai University of Traditional Chinese Medicine, Shanghai, China; Center for Drug Clinical Research, Shanghai University of Traditional Chinese Medicine, Shanghai, China; Center for Drug Clinical Research, Shanghai University of Traditional Chinese Medicine, Shanghai, China; Center for Drug Clinical Research, Shanghai University of Traditional Chinese Medicine, Shanghai, China; Center for Drug Clinical Research, Shanghai University of Traditional Chinese Medicine, Shanghai, China; Center for Drug Clinical Research, Shanghai University of Traditional Chinese Medicine, Shanghai, China; Center for Drug Clinical Research, Shanghai University of Traditional Chinese Medicine, Shanghai, China; Center for Drug Clinical Research, Shanghai University of Traditional Chinese Medicine, Shanghai, China; State Key Laboratory of Integration and Innovation of Classic Formula and Modern Chinese Medicine, Shanghai University of Traditional Chinese Medicine, Shanghai, China

**Keywords:** treatment-resistant depression, model-based meta-analysis, efficacy evaluation, modeling and simulation

## Abstract

**Objective:**

This study aims to quantitatively evaluate the efficacy and safety of various treatment regimens for treatment-resistant depression (TRD) across oral, intravenous, and intranasal routes to inform clinical guidelines.

**Methods:**

A systematic review identified randomized controlled trials on TRD, with efficacy measured by changes in the Montgomery–Åsberg Depression Rating Scale (MADRS). We developed pharmacodynamic and covariate models for different administration routes, using Monte Carlo simulations to estimate efficacy distribution. Dropout and adverse event–related dropout rates were analyzed via single-arm meta-analysis.

**Results:**

Involving 22 studies with 56 treatment arms and 3059 patients, our findings suggest combination therapies outperform monotherapy, achieving an additional 6.5% reduction in MADRS scores over 12 weeks. The most effective combinations were olanzapine with fluoxetine and quetiapine with selective serotonin reuptake inhibitors/ selective serotonin and norepinephrine reuptake inhibitors. Injectable treatments, particularly ayahuasca, produced rapid effects, with a 77% reduction in MADRS scores at 15 days. Intranasal treatments reached efficacy sooner than oral ones, with 28-day efficacy similar to the 12-week efficacy of the olanzapine–fluoxetine combination. Dropout rates due to adverse events were similar across methods (4.5%–5.2%), but total dropouts were highest for oral (17.9%) and lowest for intranasal routes (10.6%). Additionally, there was considerable variation in the incidence of headache, dizziness, and nausea across different administration routes.

**Conclusions:**

The quantitative evaluation of 22 TRD treatments illuminates key pharmacodynamic parameters, bolstering the development of clinical guidelines and aiding the design of clinical trials and medical decision-making.

**Significance Statement**

This study provides a systematic and quantitative assessment of 22 treatment options for treatment-resistant depression, encompassing oral, intravenous, and intranasal administration routes. By identifying key pharmacodynamic parameters, including maximum efficacy and onset time, our findings offer measured insights into the comparative benefits and limitations of each treatment modality.

## INTRODUCTION

According to data from the World Health Organization in 2023, the global prevalence of depression exceeds 280 million individuals.^1^In 2020 alone, the coronavirus disease 2019 (COVID-19) pandemic contributed to a 27.6%^2^ increase in the incidence of major depressive disorder (MDD) worldwide. Approximately 20%–30% of patients diagnosed with depression do not respond to standard antidepressant treatments or develop resistance, a condition known as treatment-resistant depression (TRD). The definition of TRD can vary across different studies, and there is no single universally accepted standard. Consequently, we have chosen to utilize the definitions provided by the US Food and Drug Administration (FDA) and the European Medicines Agency (EMA) to discuss TRD more consistently. Both the FDA and the EMA describe TRD as failing to achieve efficacy after at least 2 different antidepressant treatments, administered at adequate doses and for a sufficient duration, where the patient has maintained good treatment adherence.^3,4^ Specifically, a nonresponse is characterized as less than a 50% reduction in depression scores as measured by standardized tools such as the Hamilton Depression Scale (HAMD) or the Montgomery–Åsberg Depression Rating Scale (MADRS).^5^ Compared to patients with general depression, those with TRD often have an earlier age of onset, longer disease duration, higher recurrence rates, more severe impairment of social and executive functions, and significant dysregulation of immune system function. Additionally, the disability and suicide rates among patients with TRD are significantly higher than those with general depression,^6^ and the treatment costs and disease burden are substantially greater, making the improvement of treatment methods for TRD critically important.

Currently, the FDA has approved over 30 medications for the treatment of MDD, which are also applicable to TRD.^7^ These include atypical antidepressants, tricyclic antidepressants (TCAs),^8^ monoamine oxidase inhibitors (MAOIs),^8,9^ selective serotonin reuptake inhibitors (SSRIs),^6,10^ selective serotonin and norepinephrine reuptake inhibitors (SNRIs),^10^ norepinephrine and dopamine reuptake inhibitors,^11^ and N-methyl-D-aspartate (NMDA) receptor antagonists^12^ like ketamine and esketamine, which have been a recent focus of research. Due to the specificity and complexity of TRD, monotherapy often fails to achieve desired outcomes, hence combination therapy has become a clinical trend in treating TRD.^13^ Combination therapy primarily includes two approaches: combining different antidepressants^14-16^ or pairing an antidepressant with an augmenting agent,^17–20^ such as atypical antipsychotics, anxiolytics, mood stabilizers, or anticonvulsants. However, there is currently a lack of systematic research comparing the efficacy and safety of monotherapy versus combination therapy, including a comparison of different routes of administration (oral, intravenous, nasal), which is significant for guiding rational clinical medication practices.

This study aims to systematically integrate existing literature data to assess the pharmacological treatment strategies for TRD. By constructing a pharmacodynamic model,^21,22^ this study will quantitatively analyze the efficacy characteristics (including onset of action and maximum effect) and influencing factors of various TRD medication regimens. Given the differences in absorption rates, bioavailability, and patient tolerability associated with different routes of administration, significant variations in efficacy and safety outcomes are anticipated. Consequently, this study will compare the efficacy and safety of three administration routes—oral, intravenous, and nasal spray—to allow physicians to customize treatment plans tailored to the specific conditions, therapeutic requirements, and adherence preferences of individual patients.

## METHODS

### Research Criteria and Eligibility

This study systematically searched four major public databases: PubMed, Embase, Cochrane Library, and PsycINFO, covering a period from the inception of each database until January 24, 2023. The detailed search strategies are listed in Supplementary Method 1. We obtained the data for this study from the literature, and the ethics committee waived the need for ethical approval.

The inclusion criteria for the literature in this study were as follows: (1) participants aged between 18 and 65 years diagnosed with unipolar TRD; (2) participants without concurrent diagnoses of other comorbid diseases during the study period; (3) studies reported were randomized controlled trials (RCTs) of pharmacological interventions; (4) studies included reported changes in the MADRS scores from baseline.

The selection of the MADRS as the efficacy evaluation index was primarily based on the findings from a preliminary literature review. Studies have shown that the MADRS is used more frequently as the primary outcome in clinical trials of pharmacotherapy for TRD. In contrast, the HAMD is used less frequently due to its greater emphasis on the somatic symptoms of depression, which could lead to decreased HAMD scores with the improvement of certain somatic symptoms, even if the core symptoms of depression have not significantly abated.^23,24^ Additionally, the existence of multiple versions of the HAMD (such as the 17-item, 21-item, 22-item, 24-item, and 31-item scales) and the heterogeneity among these versions also contributed to the decision to exclude clinical studies that solely reported HAMD scores from consideration in this research.

The following exclusion criteria were established for the literature in this study: (1) Studies involving participants diagnosed with bipolar disorder or other psychiatric conditions, including but not limited to post-traumatic stress disorder, trauma-related stress disorders, prepartum or postpartum depression, vascular dementia, etc.; (2) Studies where the intervention included more than just pharmacotherapy, such as those involving psychological therapy or physical stimulation treatments; (3) Meta-analyses that combined the results of multiple clinical trials; (4) Studies with a sample size of fewer than 10 participants.

### Data Extraction and Quality Assessment

Information extracted from the included literature comprised the following: trial characteristics (including dosage, group allocation, sample size, duration of treatment, whether it was a placebo-controlled trial, and whether it was a multicenter clinical trial, etc.), participant characteristics (including age, weight, body mass index [BMI], gender ratio, ethnicity, region, time since diagnosis of depression, age at diagnosis of MDD, duration of illness, prior treatment history, number of depressive episodes, history of at least 1 suicide attempt, whether they were inpatients, and baseline scores, etc.), and trial outcomes (including changes in MADRS scores from baseline at each observation time point, overall dropout rates, dropout rates due to adverse events, and adverse event rates, etc.). If efficacy data were presented graphically, the Engauge Digitizer software (version 4.1) was used for data extraction. Errors in the data extraction process were maintained below 2%; if the error exceeded 2%, data extraction was repeated. The final data were the average of 2 extractions. Data extraction was conducted independently by 2 researchers (Y.F and Y.L), with a third resolving any discrepancies (L.L).

The quality of the included literature was assessed using the Cochrane Risk of Bias tool 2.0,^25^ which evaluates the generation of random sequences, allocation concealment, blinding of participants and personnel, blinding of outcome assessment, incomplete outcome data, selective reporting, and other potential biases. Each domain was classified as “low risk,” “high risk,” or “unclear risk.” The standards for defining the quality of literature were as follows: (1) low-quality studies: those with “high risk” of bias in either random sequence generation or allocation concealment; (2) high-quality studies: those with “low risk” of bias in both random sequence generation and allocation concealment and “low risk” or “unclear risk” in all other domains; (3) medium-quality studies: those that did not meet the criteria for either low- or high-quality studies.

### Model Development

A pharmacodynamic model was established using the change in MADRS scores from baseline as the efficacy indicator. This study focused on examining 3 structural models: the Sigmoid *E*_max_ model, the exponential model, and the Beta-turn model, to describe the time course of changes in MADRS scores from baseline. The specific mathematical expressions are presented in Equations 1–3.


$$\begin{array}{l} E=-\frac{E_{max}\times Time^{\gamma }}{E{T_{50}}^{\gamma }+Time^{\gamma }} \end{array}$$ (1)

$$\begin{array}{l} E=-E_{max}\times \left(1-e^{-k\times time}\right) \end{array}$$ (2)

$$\begin{array}{l} E=-E_{max}\times \left(e^{-k_{of\;f}\times time}-e^{-k_{on}\times time}\right) \end{array}$$ (3)

In the aforementioned equations, the pharmacodynamic parameter *E*_max_ represents the maximum effect, reflecting the maximal response achievable by the drug; Time denotes the observation time, measured in days. In Equation 1, *ET*_50_ is the time required to achieve 50% of the maximum effect, indicating the onset speed of the drug; *γ* is the steepness coefficient, which controls the shape of the efficacy curve. In Equation 2, *k* is the rate of onset, and the time to reach 50% of the maximum effect can be calculated using the following formula: *ET*_50_ = 0.693/*k*. Equation 3 can be used to describe the rebound phenomenon of drug efficacy, where *K*_on_ is the rate constant for the onset of drug effect, and *K*_off_ is the rate constant for the offset of drug effect. The data were fitted using a nonlinear mixed-effects model, and the optimal structural model was selected based on the objective function value (OFV), model diagnostic plots, and the precision of the parameter estimates.

Interstudy variability, which characterizes the differences in drug effects observed across various studies, was incorporated into the pharmacodynamic model as an exponential term to account for between-study differences in the model parameters, as delineated in Equation 4. Unexplained variability, not captured by the interstudy variability, was modeled as residual error using an additive error model, which is detailed in Equation 5.


$$\begin{array}{l} P_{i}=P_{typical}\times e^{\eta_{i}} \end{array}$$ (4)

$$\begin{array}{l} E_{obs,i,j}=E_{pred,i,j}+\frac{\varepsilon_{i,j}}{\sqrt{N_{i,j}}} \end{array}$$ (5)

In Equation 4, *P*_typical_ denotes the typical value for the model parameter. The term *η*_*i*_ represents the interstudy variability for the model parameter, which is modeled as a normally distributed random effect with a mean of zero and a variance of *ω*^2^. In Equation 5, *E*_obs,*i,j*_ indicates the observed drug effect at the *j*^th^ time point in the *i*^th^ study, while *E*_pred,*i,*j_ denotes the predicted drug effect at the same time point. The residual variability, *ε*_*i,j*_, captures the unexplained deviation at the *j*^th^ time point in the *i*^th^ study. The term *N*_*i,j*_ reflects the sample size at the *j*^th^ time point in the *i*^th^ study, which adjusts *ε*_*i,j*_ by the inverse square root, implying that larger sample sizes are associated with reduced residual errors. The residual error, *ε*_*i,j*_, is assumed to be normally distributed with a mean of zero and a variance of *σ*^2^.

After establishing the base model, potential covariates that may influence the pharmacodynamic parameters were explored, including subject age, weight, BMI, proportion of females, proportion of Caucasians, concomitant medication use, inpatient status, and baseline MADRS scores. In this study, covariate analysis was only conducted on variables with a missing rate of less than 30%. Missing covariate data were imputed conservatively using the median value. The method of incorporating continuous covariates is presented in Equations 4–6, while the approach for binary covariates is detailed in Equation 7.


$$\begin{array}{l} P_{i}=P_{typical}+\left(COV-COV_{median}\right)\times \theta_{cov} \end{array}$$ (6)

$$\begin{array}{l} P_{i}=P_{typical}\times {\left(COV/COV_{median}\right)}^{\theta_{cov}} \end{array}$$ (7)

$$\begin{array}{l} P_{i}=P_{typical}\times e^{\left(COV-COVmedian\right)\times \theta_{cov}}\; \end{array}$$ (8)

$$\begin{array}{l} P_{i}=P_{typical}+COV\times \theta_{cov} \end{array}$$ (9)

In Equations 6–9, *P*_*i*_ represents the model parameter at different levels of the covariate, while *P*_typical_ is the typical value of the model parameter, defined as the parameter value when a binary variable is set to 0, or when a continuous variable is at its median value. COV denotes the covariate value, COV_median_ is the median value of the covariate, and *θ*_cov_ is the correction coefficient for the effect of the covariate on the model parameter.

The influence of each covariate on the model parameters was scrutinized individually. A covariate was considered to significantly affect the parameters if inclusion resulted in a decrease of the model’s OFV that exceeded 3.84, which is the critical value for a chi-square distribution with 1 degree of freedom at a significance level of *P* < .05. Subsequently, covariates identified as significant through this univariate analysis were subjected to multivariate analysis using forward selection and backward elimination techniques to ascertain the definitive set of covariates for inclusion in the model. The criterion for inclusion in the forward selection step was set at an OFV decrease of 3.84 (*P* < .05), whereas for retention in the backward elimination step, the threshold was an OFV decrease of 6.63 (*P* < .01).

### Model Evaluation

The performance of the final model was rigorously assessed using a variety of metrics,^26^ such as the standard error of the estimated parameters, OFV, goodness-of-fit plots (GOF), Bootstrap analysis, visual predictive checks (VPC), and an evaluation of clinical plausibility. Bootstrap analysis was employed to ascertain model robustness by comparing the parameter estimates obtained from 1000 bootstrap resamples to those derived from the original dataset. VPC were conducted by simulating 1000 datasets from the model to generate the 97.5th, 50th, and 2.5th percentiles of the predicted drug efficacy distribution. These simulated percentiles were then plotted alongside the observed efficacy data to assess the model’s predictive accuracy.

### Model Simulation

Based on the final model, pharmacodynamic parameter estimates for each treatment group, along with their standard errors, were calculated using Bayesian feedback. When a pharmacodynamic parameter was significantly influenced by a covariate, covariate-adjusted parameter estimates were obtained through the inverse operation of the covariate model to facilitate the comparability of pharmacodynamic parameters across different drugs under the same conditions. A random-effects model within a single-arm meta-analysis framework was employed to aggregate the covariate-adjusted pharmacodynamic parameters from each treatment group according to the type of treatment regimen. This process yielded the distribution of pharmacodynamic parameters for different treatment regimens, providing point estimates and their 95% confidence intervals (CIs). Subsequently, parameter values were randomly sampled from the distribution of pharmacodynamic parameters for each treatment regimen to calculate drug efficacy at various time points. This simulation was repeated 10 000 times to derive the median drug efficacy and its 95% CI at different time points for each treatment regimen.

### Analysis of Dropout Rates and Adverse Events

Most of the studies included in our analysis report only the dropout rates and incidence of adverse events at the endpoint, preventing us from establishing a time-course model for these outcomes. To address this, we conducted a single-arm meta-analysis using a random-effects model to compare the dropout rates and adverse events across 3 different routes of administration.

### Software

Model construction and individual pharmacodynamic parameter estimation using Bayesian feedback were performed with NONMEM 7.3 (ICON Development Solutions). All model parameter estimations were conducted using the First Order Conditional Estimation method with Interaction (FOCE-I) available in NONMEM. Model simulations, single-arm meta-analysis, and graphical representations were carried out using R version 4.2.1 (The R Foundation for Statistical Computing).

## RESULT

### Characteristics of the Included Studies

A total of 3505 articles were retrieved from the literature search. Following abstract and full-text screening, 22 articles met the inclusion criteria (see Supplementary Materials for the detailed screening process). These 22 articles included 56 arms comprising 3059 subjects for analysis. Out of these arms, 22 were oral administration regimens, which consisted of conventional oral antidepressants used alone or in combination with quetiapine, lithium, lamotrigine, metyrapone, riluzole, etc.; 22 arms were intravenous administration regimens, including injections of Ketamine, Esketamine, Ayahuasca, as well as combinations with oral clonidine or lithium; and 12 arms involved intranasal administration regimens, such as nasal sprays of esketamine or ketamine in combination with oral antidepressants. Details of the included dosing regimens are provided in Table 1.

**Table 1.** List of treatment regimens included in the study.

Regimens	Number of arms	Sample size
**Oral administration**	22	2100
SSRIs/SNRIs	4	390
Other antidepressants^a^	4	212
Quetiapine	1	225
Fluoxetine	2	148
Olanzapine	1	62
Venlafaxine	1	59
Olanzapine + Fluoxetine	3	390
Quetiapine + SSRIs/SNRIs	1	229
Lithium + SSRIs/SNRIs	1	221
Riluzole + SSRIs/SNRIs	2	64
Metyrapone + other antidepressants^a^	1	83
Lamotrigine + other antidepressants^a^	1	17
**Intravenous administration**	22	383
Ayahuasca	1	17
Esketamine	3	31
Ketamine	13	257
MIJ821	2	40
Ketamine + Clonidine	2	20
Ketamine + Lithium	1	18
**Intranasal administration**	12	676
Esketamine + SSRI/SNRI	4	475
Esketamine + other antidepressants^a^	6	156
Ketamine + Brexpiprazole	1	25
Ketamine	1	26

Abbreviations: SNRI, serotonin and norepinephrine reuptake inhibitor; SSRI, selective serotonin reuptake inhibitor.

^a^Other antidepressants: other oral antidepressants that were not explicitly described in the included study or that were not in the SSRIs/SNRIs class.

The mean age of the participants ranged from 26 to 52.3 years (median of 44.2 years), with a female representation of 34.1%–82.4% (median of 58.5%). The baseline MADRS scores ranged from 27.7 to 38.4 (median of 33.7). Detailed demographic baseline characteristics of the participants are presented in Table 2, while the comprehensive information of the included articles is available in Supplementary Materials.

**Table 2.** Basic characteristics of the included studies

	Overall	Oral administration	Intravenous administration	Intranasal administration
Number of regimens	22	12	6	4
Number of arms	56	22	22	12
Sample size per arm	26 (10–243)	81 (17–243)	17 (10–48)	40.5 (11–126)
Age, years (min–max)	44.2 (26–52.3)	43.4 (26–47.6)	45.6 (39.7–52.3)	43.7 (36.8–49.8)
Female, % (min–max)	58.5 (34.1–82.4)	61.3 (36–82.4)	58.3 (34.1–80)	56 (40–81.8)
White, % (min–max)	78.8 (7.1–100)	83.3 (7.1–100)	79.1 (35.6–100)	71.0 (9.5–93)
Baseline MADRS* score (min–max)	33.7 (27.7–38.4)	32.2 (27.7–37.7)	33.4 (30.8–36.9)	35.6 (31.3–38.4)

Abbreviation: MADRS*, Montgomery–Åsberg Depression Rating Scale.

Within the 22 articles, 8 (38.1%) were assessed as high quality, 11 (47.6%) as moderate quality, and 3 (14.3%) as low quality. Details on the risk of bias assessment for the studies are provided in Supplementary Materials.

### Model Establishment and Evaluation

Exploratory data analysis (Figure S2) revealed substantial differences in the change over time of MADRS scores from baseline among different administration routes. This study will conduct separate modeling analyses for the efficacy data of the different administration routes. In terms of base model selection, the Exponential model was ultimately chosen to describe the time–effect relationship of the oral administration regimen, while the Sigmoid *E*_max_ model was selected for regimens involving intravenous and intranasal administration. The rationale for the selection of the base models is detailed in Supplementary Appendix.

During covariate screening, it was discovered that the baseline MADRS score significantly influenced the *E*_max_ value of the oral administration regimen. The specific covariate equations are presented in Equations 8. Details of the covariate screening are provided in the Supplementary Material, and the estimated parameters of the final models can be found in Table 3.

**Table 3.** Parameter estimates of the final model.

	Oral administration model		Intravenous administration model		Intranasal administration model	
	Final model	Bootstrap (955/1000*)	Final model	Bootstrap (996/1000*)	Final model	Bootstrap (869/1000*)
	Estimates (RSE,%)	Median (95%PI)	Estimates (RSE,%)	Median (95% PI)	Estimates (RSE, %)	Median (95% PI)
Structure parameters						
*θ*_*E*__max_	11.0 (11.7)	11.9 (8.14–19.2)	16.9 (5.7)	16.9 (15.1–18.8)	14.4 (6.4)	14.2 (10–15.6)
*θ*_*ET*__50_	\	\	0.2, Fixed	–	1.58, Fixed	–
*θ*_*k*_	0.0529 (32.9)	0.0497(0.0199–1.45)	\	\	\	\
*θ*_seline_ on *E*_max_	3.49 (21.1)	3.19(004–5.44)	\	\	\	\
Interstudy variability						
*η*_*E*__max_	3.27 (19.7)	3.52 (0.01–5.53)	0.245(19.4)	16.9(15.1–18.8)	0.088 (59.3)	0.088 (0–0.361)
η_k_,%	0.342 (35.7)	0.512(0.003–3.53)	\	\	\	\
Residual error						
*ε*	12.45 (29.2)	9.46 (3.16–29.0)	12.41 (25.3)	9.67 (6.68–11.8)	25.16 (15.0)	24.86 (18.52–30.73)

The parameter *θ*_*E*max_ represents the maximal effect of the drug, while *θ*_*ET*50_ denotes the time required to achieve 50% of *E*_max_. The parameter *θ*_*k*_ characterizes the rate of onset of the drug’s effect. The term *θ*_Baseline_ on *E*_max_ refers to the covariate parameter that assesses the impact of baseline values of MADRS on *E*_max_. The symbol *η* signifies the interstudy variability in the model parameters, and *ε* denotes the residual error. The abbreviation CI stands for confidence interval, PI stands for percentile interval, and RSE represents the relative standard error.

^a^The convergence success rates for 1000 times of the Bootstrap method were 95.5% for the oral administration model, 99.6% for the intravenous administration model, and 86.9% for the intranasal administration model.


$$\begin{array}{l} E_{max,i}^{Oral}=11\times {(\frac{Baseline_{i}}{30.7})}^{3.49} \end{array}$$ (10)

**Figure 1.**
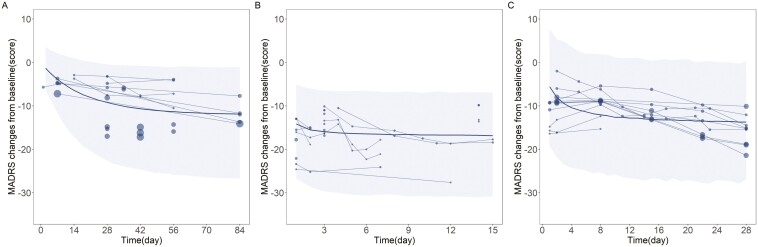
Visual predictive check of the final model for oral administration (A), intravenous administration (B) and intranasal administration (C)

In Equation 10, $E_{max,i}^{Oral}$ represents the *E*_max_ value for the *i*th treatment group of oral administration regimen, Baseline_*i*_ is the baseline MADRS score for the *i*th treatment group, 30.7 is the median baseline MADRS score for subjects in the oral administration regimen, and 3.49 is the correction coefficient for the influence of the MADRS baseline on the *E*_max_ parameter. As the baseline MADRS score increases from 30 to 35, the *E*_max_ parameter value for the oral administration regimen increases from 10.1 to 17.4. The goodness-of-fit plots (Figure S4) indicate a strong correlation between observed values and predicted values, with the trend line closely approximating the diagonal, demonstrating that the model fits the observed data well. From the conditional weighted residuals (CWRES) plot, it is evident that the majority of CWRES values are distributed between ±6, evenly scattered above and below the abscissa, with no apparent bias. The VPC graph of the final model (Figure 1) shows that most of the observed data points fall within the 95% CI of the model-predicted values, suggesting the model has good predictive performance. The median parameter estimates obtained by the bootstrap method are very close to the final model’s parameter estimates (Table 3), indicating robustness in the model parameter estimation.

### Comparison of Typical Efficacy of Oral Administration Regimens

The influence of baseline MADRS scores on the maximum effect (*E*_max_) of oral administration regimens was significant. To reduce heterogeneity between trials, MADRS baseline scores were adjusted to the median level of 30.7 for comparative efficacy analysis across different regimens.

**Figure 2.**
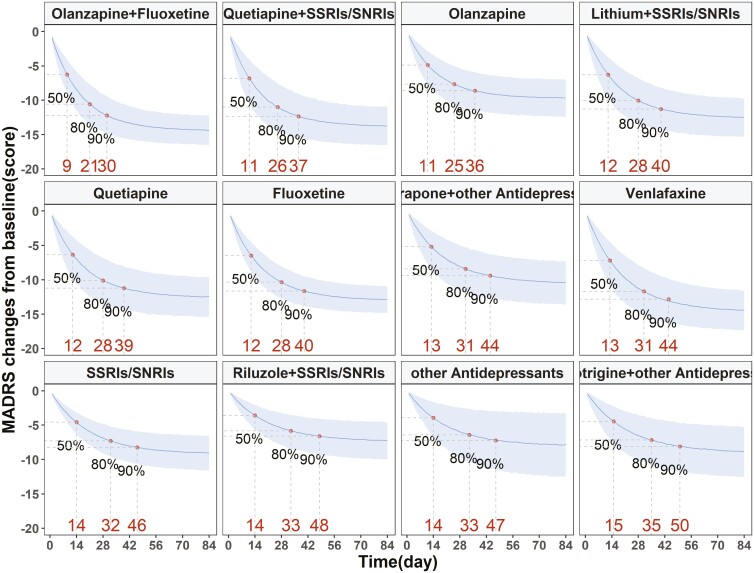
Typical time-course curves for various oral administration regimens

This study included 12 oral administration regimens, comprising 6 combination therapies and 6 monotherapies (Figure 2). Among the combination therapies, the co-administration of olanzapine with fluoxetine and quetiapine with SSRIs/SNRIs demonstrated the highest efficacy, with a reduction in MADRS scores at 12 weeks of 14.36 (95% CI: 12.18–16.55) and 13.78 (95% CI: 10.96–16.56) points, respectively. The combination of lithium with SSRIs/SNRIs and metyrapone with other antidepressants showed intermediate efficacy, with reductions in MADRS scores at 12 weeks of 12.47 (95% CI: 9.7–15.33) and 10.37 (95% CI: 7.25–13.59) points, respectively. The lowest efficacy was seen in the combination of lamotrigine with other antidepressants and riluzole with SSRIs/SNRIs, with reductions in MADRS scores at 12 weeks of 8.85 (95% CI: 5.26–12.52) and 7.29 (95% CI: 4.62–10.07) points, respectively.

Among monotherapies, venlafaxine, fluoxetine, and quetiapine showed higher efficacy, with reductions in MADRS scores at 12 weeks of 14.42 (95% CI: 11.57–17.28), 12.89 (95% CI: 10.96–14.82), and 12.43 (95% CI: 9.56-–15.35) points, respectively. Olanzapine and SSRIs/SNRIs demonstrated intermediate efficacy, with reductions in MADRS scores at 12 weeks of 9.68 (95% CI: 7.05–12.37) and 9.07 (95% CI: 6.58–11.59) points, respectively. Other antidepressants showed the lowest efficacy, with a reduction in MADRS scores at 2 weeks of 7.83 (95% CI: 3.24–12.34) points.

When olanzapine was co-administered with fluoxetine, there was an additional reduction of 4.68 points in MADRS scores at 12 weeks compared to olanzapine monotherapy. The combination of quetiapine with SSRIs/SNRIs resulted in an additional decrease of 1.35 points in MADRS scores at 12 weeks compared to Quetiapine alone. The combination of lithium with SSRIs/SNRIs and riluzole with SSRIs/SNRIs, compared to SSRIs/SNRIs monotherapy, led to an additional decrease of 3.4 points and a lesser decrease of 1.78 points in MADRS scores at 12 weeks, respectively. The co-administration of metyrapone with other antidepressants and lamotrigine with other antidepressants, compared to other antidepressant monotherapies, resulted in an additional decrease of 2.54 points and 1.02 points in MADRS scores at 12 weeks, respectively.

**Figure 3.**
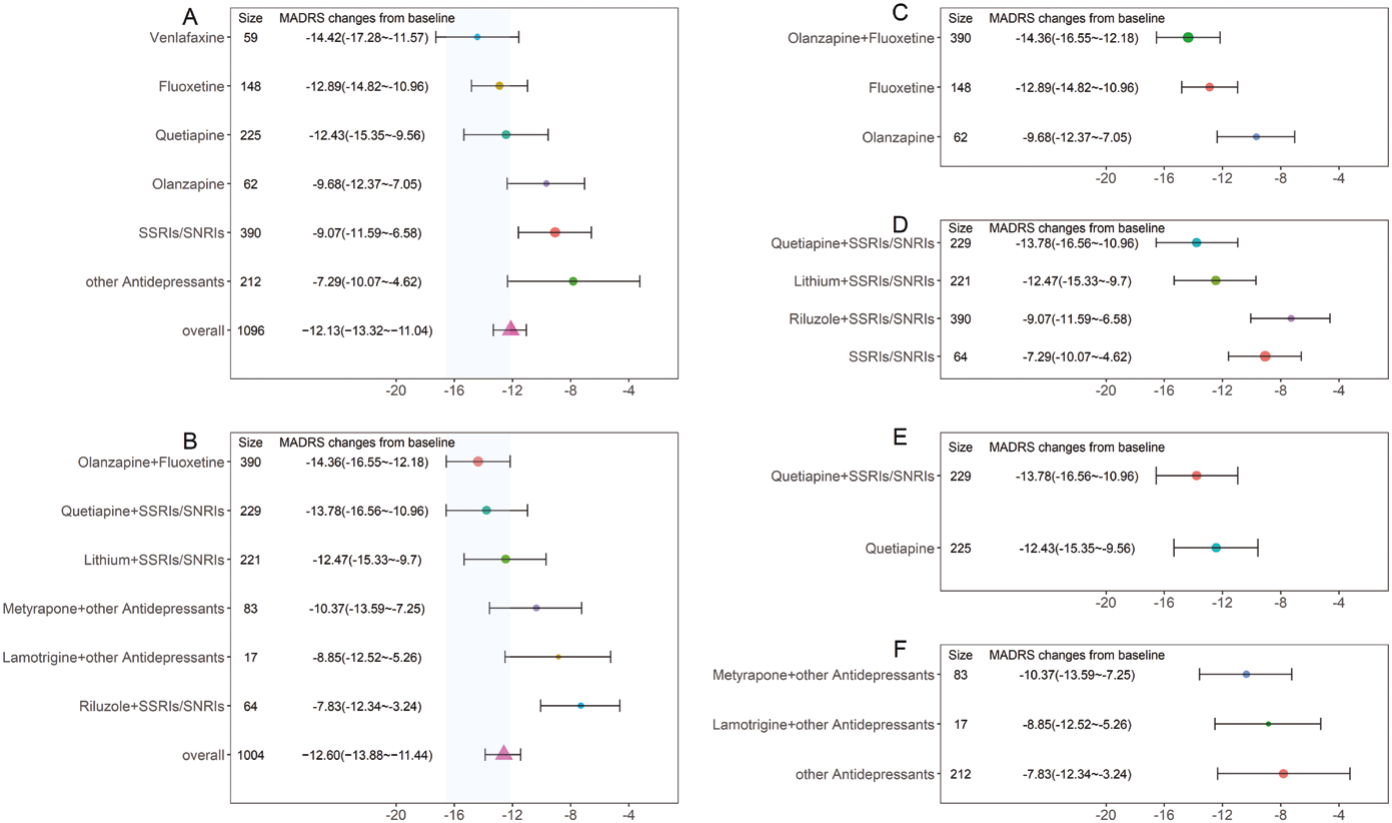
Comparison of efficacy distribution of oral administration regimen

Furthermore, this study modeled the time–effect curves (Figure 3) for 12 oral administration regimens and found that the combination of olanzapine with fluoxetine had the fastest onset of action, reaching the efficacy plateau (90% of the maximum effect) within 30 days. The onset of action for the combinations of quetiapine with SSRIs/SNRIs, lithium with SSRIs/SNRIs, and the monotherapies of olanzapine, fluoxetine, and quetiapine was comparable, reaching the efficacy plateau within 40 days. The combinations of metyrapone with other antidepressants, riluzole with SSRIs/SNRIs, and lamotrigine with other antidepressants, and had a slower onset of action, requiring more than 44 days to reach the efficacy plateau.

### Comparison of Typical Efficacy of Intravenous Regimens

**Figure 4.**
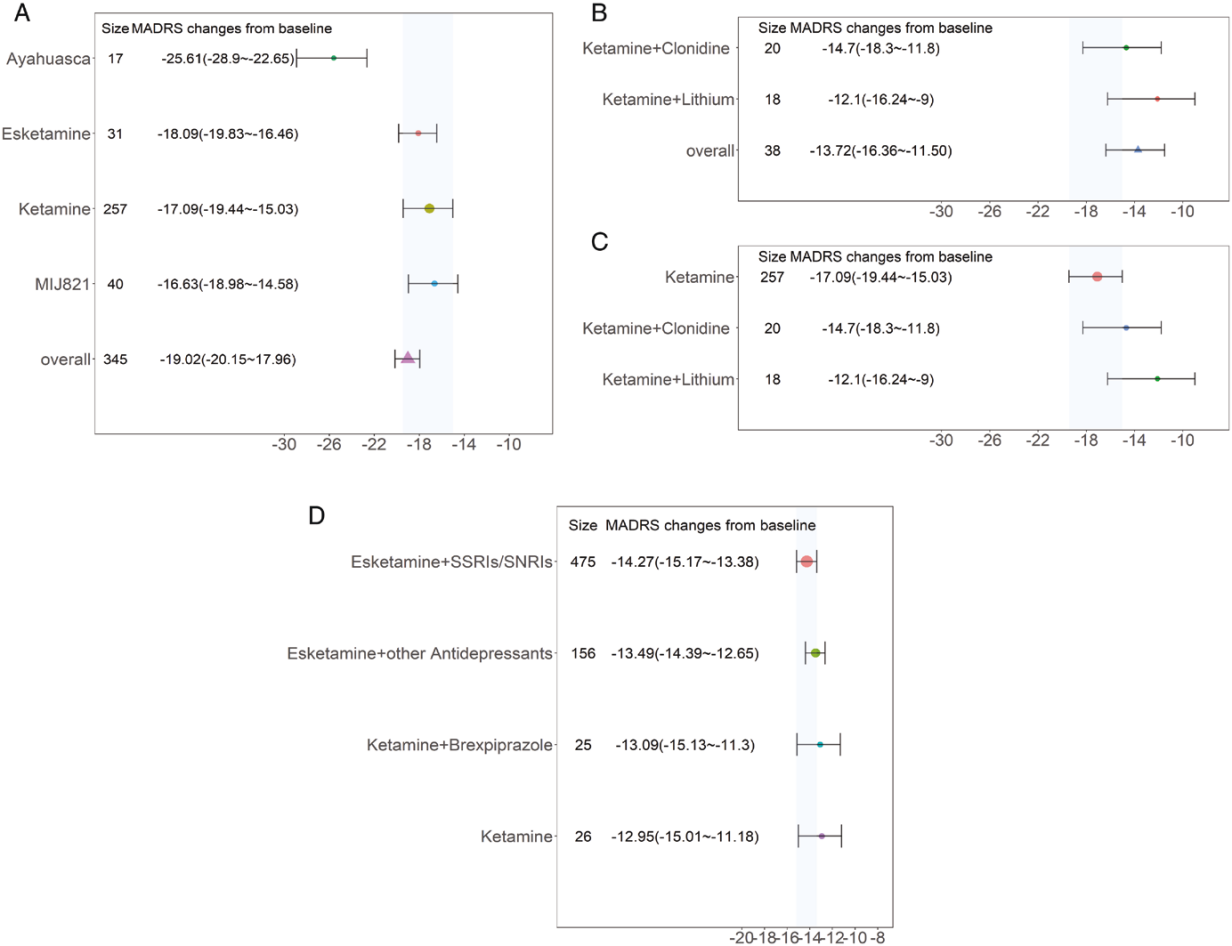
Efficacy distribution of intravenous and intranasal administration regimens

This study included 6 administration regimens with intravenous formulations: ayahuasca, esketamine, ketamine, MIJ821, ketamine combined with clonidine, and ketamine combined with lithium. The results indicated (Figure 4A-C) that the onset of action for the intravenous formulation regimens was very rapid, with the efficacy plateau (90% of maximum effect) being reached by the second day of medication. Among these, ayahuasca exhibited the most optimal efficacy performance, yielding a reduction of 25.6 points (95% CI: 22.7–28.9) in MADRS scores at 15 days, significantly surpassing other administration regimens. Ketamine alone resulted in a decrease of 17.1 points (95% CI: 15.0–19.4) in MADRS scores at 15 days. Additionally, it was found that the efficacy of ketamine in combination with clonidine or lithium did not enhance the treatment effect compared to ketamine monotherapy. The efficacy of esketamine and MIJ821 was also comparable to that of ketamine.

### Comparison of Typical Efficacy of Intranasal Administration Regimens

This study included 4 intranasal administration regimens: nasal spray esketamine in combination with oral SSRIs/SNRIs medication, nasal spray esketamine with other antidepressants, nasal spray ketamine combined with oral brexpiprazole, and nasal spray ketamine as monotherapy. The results indicated that the intranasal regimens reached an efficacy plateau (90% of the maximum effect) by day 15 post-administration. The efficacy of the 4 regimens was similar (Figure 4D); for instance, the esketamine + SSRIs/SNRIs regimen resulted in a reduction of 14.27 points (95% CI: 13.38–15.17) in the MADRS score by day 28 of the medication course.

### Analysis of Dropout Rates and Adverse Events

A meta-analysis was conducted to compare the dropout rates and the rates of dropout due to adverse events across 3 different routes of administration. The results indicated that the oral administration regimen had a dropout rate of 17.9% (95% CI: 13.7%–22.2%) and a rate of dropout due to adverse events of 5.2% (95% CI: 3.1%–7.3%). The regimen involving injections had a dropout rate of 15.6% (95% CI: 8.8%–22.5%) and a rate of dropout due to adverse events of 4.5% (95% CI: 0.4%–8.7%). The intranasal administration regimen had a dropout rate of 10.6% (95% CI: 6.9%–14.2%) and a rate of dropout due to adverse events of 4.5% (95% CI: 2.2%–6.8%).

**Figure 5.**
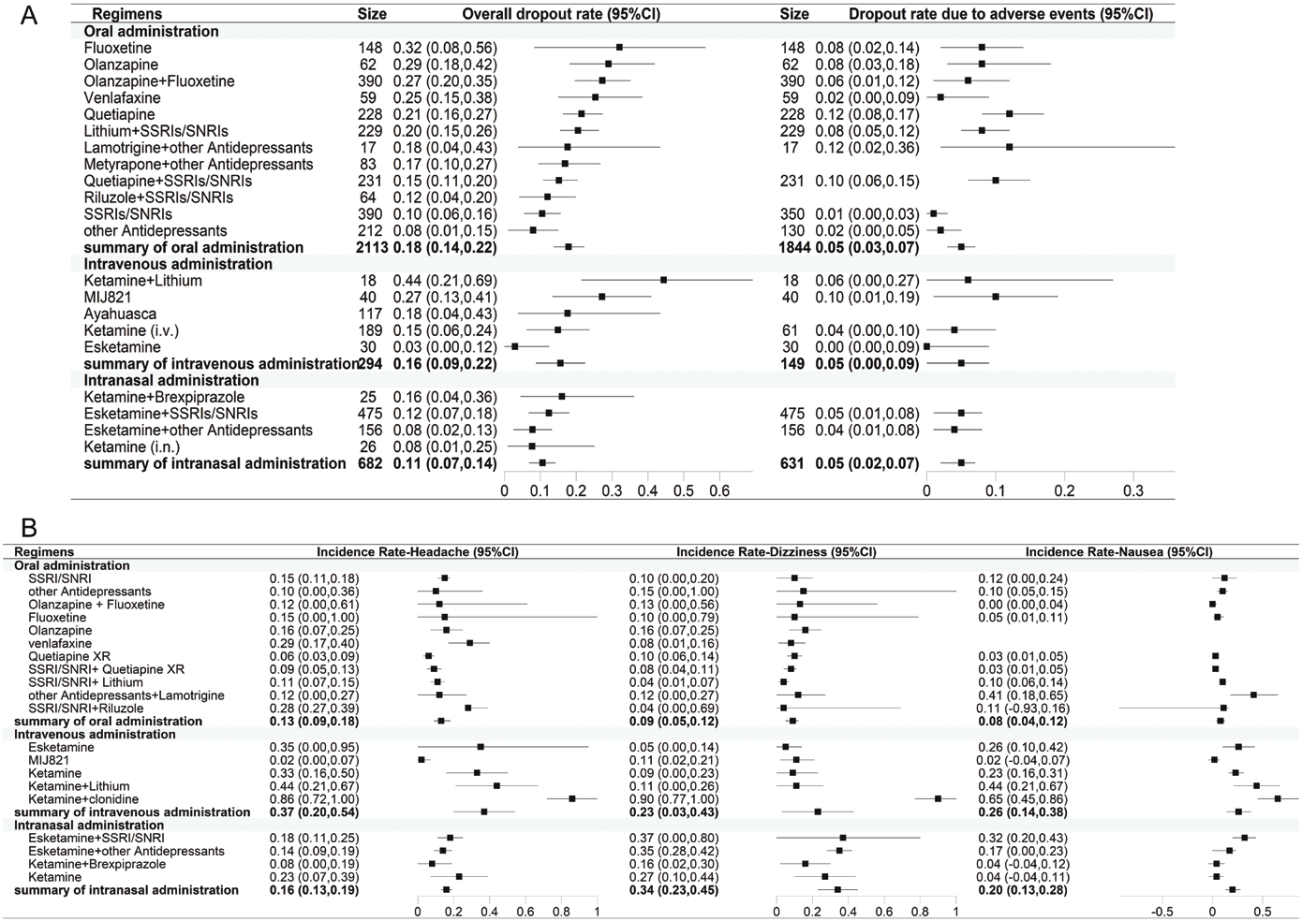
Distribution (A) dropout rate; (B) incidence of adverse events.

The literature frequently reports adverse events including headache, dizziness, and nausea. The incidence rates for these events vary among different administration routes (Figure 5). Specifically, for headaches, the oral administration route has an incidence rate of 13% (95% CI: 9%–18%), the injection route 37% (95% CI: 20%–54%), and the intranasal administration route 16% (95% CI: 13%–19%). Regarding dizziness, the rates are 9% (95% CI: 5%–12%) for oral administration, 23% (95% CI: 3%–43%) for injections, and 34% (95% CI: 23%–45%) for intranasal administration. For nausea, the incidence rates are 8% (95% CI: 4%–12%) for oral administration, 26% (95% CI: 14%–38%) for injections, and 20% (95% CI: 13%–28%) for intranasal administration.

## DISCUSSION

This study, based on an extensive compilation of literature data, developed pharmacodynamic models for three routes of administration—oral, injection, and intranasal—using the change from baseline in MADRS scores as an efficacy endpoint. Building on this foundation, the study conducted a quantitative evaluation and comparison of 22 medical treatment regimens currently used for the treatment of TRD. The findings of this study indicate that within oral administration regimens, the baseline MADRS score is a determinant of drug efficacy; higher baseline levels are associated with better treatment outcomes. Therefore, when making cross-study comparisons, it is essential to adjust for the heterogeneity of MADRS baseline scores.

This study demonstrates that, in oral administration regimens, combination therapy generally exhibits higher efficacy than monotherapy, with an additional reduction of 1.87 points on the MADRS score at 12 weeks. Given that the median baseline value of MADRS is 30.7, this corresponds to a further decrease of 6.5%. Among combination therapies, common pairings involve SSRIs/SNRIs antidepressants with olanzapine, quetiapine, and lithium. Notably, the combination of the SSRIs/SNRIs representative drug fluoxetine with olanzapine showed optimal efficacy, with the fastest onset of action, leading to a 46.8% reduction in MADRS scores at 12 weeks. Olanzapine, a widely used atypical antipsychotic, primarily blocks dopamine D2 receptors and serotonin 5-HT2A receptors, and also exhibits affinity for various other receptors such as adenosine, histamine, and muscarinic receptors.^27^ Its broad receptor activity contributes to the regulation of mood, cognition, sleep, and appetite and can be used for combination therapy in TRD, especially in conjunction with fluoxetine^28^ (OFC combination therapy), marketed under the trade name Symbyax, which has been approved by the FDA for the treatment of TRD in adults.^29^ Quetiapine, another atypical antipsychotic, blocks dopamine D2 and serotonin 5-HT2 receptors and has affinity for other serotonin, norepinephrine, and histamine receptors, which can alleviate depressive symptoms and is used in the treatment of TRD.^30^ This study shows that the efficacy of quetiapine combined with SSRIs/SNRIs is similar to that of olanzapine combined with fluoxetine and superior to monotherapy with SSRIs/SNRIs or quetiapine alone. Lithium, as mood stabilizers with an incompletely understood mechanism of action that likely involves multiple neurotransmission pathways, is primarily used as an augmenting agent in combination with antidepressants for the treatment of TRD.^31,32^ This study indicates that the combination of lithium with SSRIs/SNRIs is slightly less effective than the olanzapine or quetiapine combinations, but still results in an additional 3.3-point reduction in MADRS scores over SSRIs/SNRIs monotherapy, which represents 10.7% of the baseline. Beyond these mainstream combination therapies, the study also included 3 exploratory combination protocols, such as Metyrapone with other antidepressants, lamotrigine with other antidepressants, and riluzole in combination with SSRIs/SNRIs. Among them, the combination of metyrapone with other antidepressants showed a notably increased efficacy compared to antidepressant monotherapy, with an additional 3.08-point reduction in the MADRS score. Metyrapone, by reducing the synthesis of cortisol, modulates the body’s stress response, which could have a positive impact on depressive symptoms.^33,34^ As a potential drug for the treatment of TRD, metyrapone remains in the exploratory phase, requiring more extensive clinical trials to validate its efficacy and safety.

This investigation incorporated 6 dosing regimens that included intravenous formulations, with 2 of these regimens combining intravenous injection with oral administration. The results indicated that the combined oral administration strategies did not significantly enhance efficacy compared to intravenous monotherapy. Compared to oral monotherapy, regimens including intravenous formulations were characterized by a more rapid onset of action, reaching an efficacy plateau (90% of maximum effect) by the second day of treatment, whereas oral monotherapy required more than 30 days. Moreover, when comparing the 4 monotherapy intravenous injection strategies, ayahuasca demonstrated significantly greater efficacy than other intravenous medications. This hallucinogenic compound, originating from the Amazon’s traditional medicine, consists of *Banisteriopsis caapi* vine and DMT-rich Chacruna or *Psychotria viridis*. Its mechanism involves serotonin receptor activation, chiefly at 5-HT2A, leading to swift changes in consciousness and profound visual and emotional experiences.^35^ Ayahuasca’s therapeutic and spiritual potential, particularly for TRD, has garnered rising international attention.^36,37^ This study found that ayahuasca significantly reduced depressive symptoms, decreasing the MADRS score by 25.6 points, which corresponds to a 77% reduction from baseline levels. However, scientific research on ayahuasca for the treatment of TRD is currently limited, and the conclusions of this study are based on a small-scale exploratory study. Therefore, its efficacy and safety require validation through further large-scale sample studies. Ketamine, esketamine, and MIJ821 showed similar efficacy in reducing MADRS scores, with a reduction of approximately 16.6–18.1 points by day 15, corresponding to a 51%–56% decrease from baseline levels. Ketamine, an N-methyl-D-aspartate (NMDA) receptor antagonist, mitigates excitatory neurotransmission by inhibiting glutamate’s action on NMDA receptors and may indirectly enhance the activity of neurotransmitters like serotonin, norepinephrine, and dopamine, contributing to its rapid antidepressant properties.^38^ Its use in TRD is expanding, including nasal spray and intravenous forms, yet its long-term efficacy and safety warrant further investigation.^39^ Due to its abuse potential and risk of dissociative effects, ketamine should be administered with medical oversight, and patient evaluation and monitoring are critical.^40^ Thus, while ketamine presents an innovative treatment modality for TRD, it necessitates careful application and further study to ascertain its long-term effects. Esketamine, the S-(-) enantiomer of ketamine, has a higher affinity and specificity than ketamine,^41-43^ and this study showed that esketamine can reduce the MADRS score by an additional point compared to ketamine. MIJ821 is also an NMDA receptor antagonist with a mechanism of action similar to ketamine, and small-scale exploratory studies have shown its efficacy to be comparable to that of ketamine. Research^44^ has found that MIJ821 has higher selectivity compared to ketamine, which may help to reduce the potential for drug abuse and decrease side effects. Nevertheless, the precise efficacy and safety of MIJ821 remain to be validated through additional clinical trials.

This study assessed 4 nasal spray dosing regimens using ketamine or esketamine. Results indicated no significant difference in efficacy among these nasal spray regimens, and their combination with oral antidepressants or antipsychotics did not significantly enhance therapeutic outcomes. The onset of action for nasal spray treatments was intermediate between intravenous and oral administration, reaching an efficacy plateau approximately on day 9. By day 28, the efficacy of nasal sprays was comparable to the 12-week outcomes of mainstream oral regimens, specifically the combination of olanzapine and fluoxetine. Compared to intravenouss, the development of ketamine and esketamine nasal sprays significantly improved patient compliance and quality of life, and reduced the burden on healthcare resources. Moreover, the nasal spray form facilitates more stable and controlled drug release, potentially decreasing the risk of misuse and adverse events.

The study conducted an in-depth analysis of the overall dropout rates as well as dropout rates due to adverse events across different administration regimen, comparing oral, intravenous, and nasal spray modalities. The findings showed minimal differences in dropout rates due to adverse events among the 3 methods, with all being between 4.5% and 5.2%. However, there was a greater variance in overall dropout rates: 17.9% for oral administration, 15.6% for intravenous administration, and a comparatively lower 10.6% for nasal sprays. The higher dropout rate for oral administration may be attributed to slower onset and lower efficacy, increasing the likelihood of patient discontinuation; while intravenous, despite their rapid onset and better efficacy, may have a higher dropout due to inconvenience of use. In contrast, nasal sprays had lower dropout rates due to their relatively quick onset, good efficacy, and convenience of use. The data from this study provide an important reference for sample size estimation in future clinical research and can guide the selection of clinical treatment plans.

Analysis of adverse events shows significant variation in the incidence of headache, dizziness, and nausea across different administration routes, emphasizing the need to consider these routes to minimize side effects and improve compliance. Headaches are most common with the injection route, with an incidence rate of 37%, higher than oral (13%) and intranasal (16%) routes, indicating lower tolerability for injections among headache-prone patients. Dizziness is highest with intranasal administration at 34%, exceeding oral (9%) and injection (23%) routes, likely due to its direct effects on sensitive nasal and sinus passages.^45^ Nausea peaks with injections at 26%, followed by intranasal (20%) and oral routes (8%). The more direct and rapid absorption of treatments via injections and intranasal routes may trigger stronger bodily reactions. These findings stress the importance of carefully selecting the administration route in managing TRD to balance efficacy with patient comfort and adherence.

This study has several limitations. First, it was based on an analysis of aggregated data from literature, which limited the amount and range of covariate distributions available. This restriction precluded the use of advanced methods such as machine learning to identify influencing factors. Apart from baseline MADRS scores, no other covariates were found to significantly impact the results. Second, due to the limitations of the data reported in the literature, the study could only examine covariates related to the demographic characteristics of subjects. Important potential factors affecting drug efficacy, such as key biomarkers, genetic polymorphisms, patients’ age of onset, and the duration of disease episodes, could not be assessed. Third, the number of trials examining certain regimens, especially those involving intravenous and intranasal administration routes, was limited. These studies often had small sample sizes and were exploratory in nature. As a result, the estimated efficacy distributions may be subject to significant sampling errors, necessitating cautious interpretation of the results. Fourth, the analysis was confined to short-term treatments within a 12-week period. A more comprehensive understanding of the long-term efficacy and safety of different treatment regimens requires data from extended studies. Finally, this study was restricted to English-language publications of RCTs that reported MADRS outcomes. Ten studies reporting on HAMD were not included in this analysis, which involved 7 for oral administration,^46–52^ 2 for intravenous,^53,54^ and 1 for intranasal administration regimens.^55^ Although previous studies have combined MADRS and HAMD scores following data standardization, due to the inherent heterogeneity between these measures and the uneven distribution of literature on HAMD across different study designs, a simple merger might introduce additional heterogeneity. Consequently, studies involving HAMD were excluded from this analysis, potentially leading to some degree of publication bias.

## CONCLUSIONS

This study systematically quantitatively assessed the efficacy and safety of 22 treatment options for treating TRD, covering oral administration, intravenous injection, and nasal inhalation as routes of administration. It identified key pharmacodynamic parameters such as maximum efficacy and onset time. The results not only provide quantitative support for the establishment of clinical treatment guidelines but also offer important references for the design of clinical trials and medical decision-making.

## Supplementary Material

pyaf007_suppl_Supplementary_Materials

## Data Availability

Data described in the manuscript, code book, and analytic code will be made available upon request pending application and approval.
