# Supplementary material for: Quantitative evaluation of multiple treatment regimens for treatment-resistant depression
Source: Int J Neuropsychopharmacol. 2025 Jan 25;28(2):pyaf007. doi: 10.1093/ijnp/pyaf007 (PMC11879141; doi:10.1093/ijnp/pyaf007)
Supplement: pyaf007_suppl_Supplementary_Materials [file pyaf007_suppl_supplementary_materials.docx]

**Supplementary** **Materials**

Method 1. Full literature search strategy

Table S1. Search strategy.

Table S2: The list of included studies

Table S3: Basic information of the included studies

Table S4: Risk assessment for inclusion in the studies

Figure S1. Flow chart of the literature selection

Figure S2. Data View chart

Figure S3. The summary risk of literature assessment.

Figure S4. Covariate selection plot for the oral administration model

Figure S5. Covariate selection plot for the intravenous administration model

Figure S6. Covariate selection plot for the intranasal administration model

Figure S7. The goodness-of-fit plots of oral administration model.

Figure S8. The goodness-of-fit plots of intravenous administration model

Figure S9. The goodness-of-fit plots of intranasal administration model

**Method 1. Full literature search strategy**

We conducted a comprehensive search of the PubMed/MEDLINE, Cochrane Library, EMBASE, and PsycINFO databases up to January 24, 2023, utilizing keywords such as 'refractory depression', 'difficult-to-treat depression', 'treatment-resistant depression', and other similar terms. Keywords within the same category were combined using the logical operator 'OR', while different categories were connected by 'AND'. The complete search strategy is detailed below. We imported the citations into EndNote X9, checked for duplicates, and manually removed them using EndNote X9. Two researchers were involved in the literature inclusion process; they reviewed full texts of potential studies and constructed tables for data extraction.

**Table S1. Search strategy**

| No. | Query | Results |  |
| --- | --- | --- | --- |
| PubMed | |  |  |
| #1 | **(refractory depression [Title/Abstract]) OR (difficult-to-treat depression [Title/Abstract]) OR (treatment resistant depression [Title/Abstract])** | 4561 | 24-Jan-23 |
| #2 | Filters: Clinical Trial | 608 | 24-Jan-23 |
| #3 | #1 AND #2 | 608 | 24-Jan-23 |
| Cochrane Library | |  |  |
| #1 | **(refractory depression)** **:ti,ab,kw OR (difficult-to-treat depression) :ti,ab,kw OR (treatment resistant depression) :ti,ab,kw** | 6902 | 24-Jan-23 |
| #2 | Filters: Trial | 6266 | 24-Jan-23 |
| #3 | clinical trial OR randomized controlled trial [Publication Type] | 2261 | 24-Jan-23 |
| #4 | #1 AND #2 AND #3 | 2261 | 24-Jan-23 |
| Embase | |  |  |
| #1 | **'refractory depression':ti,ab,kw OR 'difficult-to-treat depression':ti,ab,kw OR 'treatment resistant depression':ti,ab,kw** | 7062 | 24-Jan-23 |
| #2 | **([controlled clinical trial]/lim OR [randomized controlled trial]/lim)** | 566 | 24-Jan-23 |
| #3 | #1 AND #2 | 566 | 24-Jan-23 |
| PsycINFO | |  |  |
| #1 | **(refractory depression) OR (difficult-to-treat depression) OR (treatment resistant depression)** | 7922 | 24-Jan-23 |
| #2 | Filters: Clinical Trial | 670 | 24-Jan-23 |
| #3 | #1 AND #2 | 670 | 24-Jan-23 |

**Figure S1. Flow chart of the literature selection**
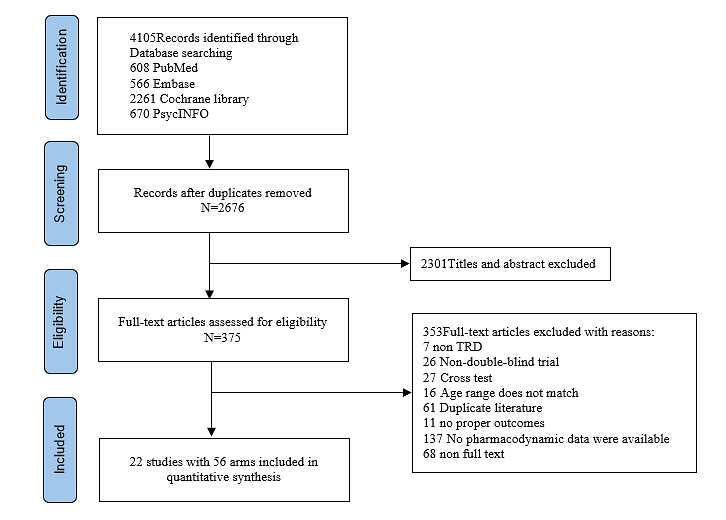


**Figure S2. Exploratory data analysis overview**


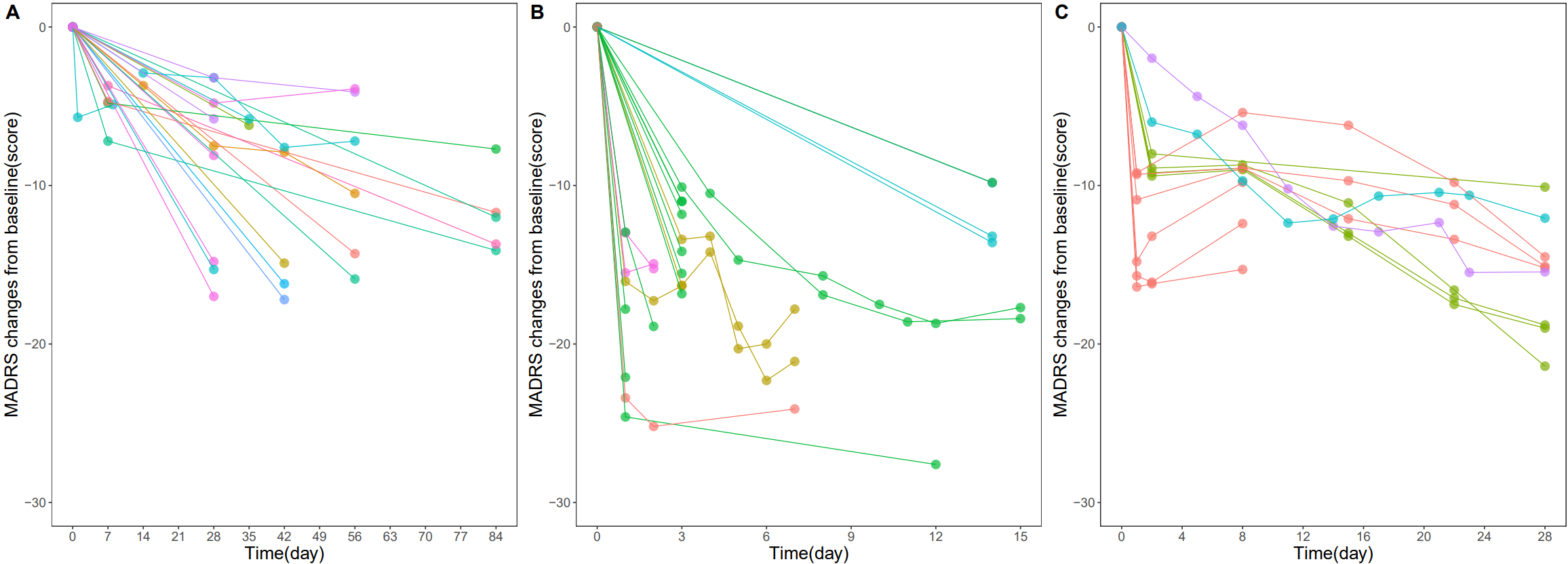


(A) Oral administration regimens, including monotherapy and combination therapy; (B) Intravenous administration regimens, including intravenous monotherapy and combinations with oral medications; (C) Intranasal administration regimens, including intranasal monotherapy and combinations with oral medications.

Data points indicate measured changes in MADRS scores from baseline, with lines connecting data from the same trial group.

**Table S2: The list of the included studies**

| ID | First author/ institution | Publication Year | Title |
| --- | --- | --- | --- |
| 01 | Vanina Popova | 2019 | Efficacy and Safety of Flexibly Dosed Esketamine Nasal Spray Combined With a Newly Initiated Oral Antidepressant in Treatment-Resistant Depression: A Randomized Double-Blind Active-Controlled Study |
| 02 | Maggie Fedgchin | 2019 | Efficacy and Safety of Fixed-Dose Esketamine Nasal Spray Combined With a New Oral Antidepressant in Treatment-Resistant Depression: Results of a Randomized, Double-Blind, Active-Controlled Study (TRANSFORM-1) |
| 04 | Janssen Research &  Development | 2018 | A Study to Evaluate the Efficacy, Pharmacokinetics, Safety and Tolerability of Flexible Doses of Intranasal Esketamine Plus an Oral Antidepressant in Adult Participants With Treatment-resistant Depression |
| 05 | Ella J. Daly | 2018 | Efficacy and Safety of Intranasal Esketamine Adjunctive to Oral Antidepressant Therapy in Treatment-Resistant Depression |
| 06 | Jaskaran B Singh | 2016 | Intravenous Esketamine in Adult Treatment-Resistant Depression: A Double-Blind, Double-Randomization, Placebo-Controlled Study |
| 07 | Nagahide Takahashi | 2021 | Efficacy and safety of fixed doses of intranasal Esketamine as an add-on therapy to Oral antidepressants in Japanese patients with treatment-resistant depression: a phase 2b randomized clinical study |
| 08 | Maurizio Fava | 2020 | Double-blind, placebo-controlled, dose-ranging trial of intravenous ketamine as adjunctive therapy in treatment-resistant depression (TRD) |
| 10 | Mu-Hong Chen | 2019 | Antidepressant and antisuicidal effects of ketamine on the functional connectivity of prefrontal cortex-related circuits in treatment-resistant depression: A double-blind, placebo-controlled, randomized, longitudinal resting fMRI study. |
| 12 | Maurizio Fava | 2017 | A Study of Brexpiprazole Plus Ketamine in Treatment-Resistant Depression (TRD) |
| 13 | Novartis Pharmaceuticals | 2018 | Proof of Concept Study Evaluating the Efficacy and Safety of MIJ821 in Patients With Treatment-resistant Depression |
| 14 | Singh, J. B. | 2016 | A Double-Blind, Randomized, Placebo-Controlled, Dose-Frequency Study of Intravenous Ketamine in Patients With Treatment-Resistant Depression. |
| 18 | James Murrough | 2013 | Ketamine Plus Lithium in Treatment-Resistant Depression |
| 19 | Rebecca B. Price | 2009 | Effects of Intravenous Ketamine on Explicit and Implicit Measures of Suicidality in Treatment-Resistant Depression |
| 23 | Lenze, E. J. | 2016 | Ninety-six hour ketamine infusion with co-administered clonidine for treatment-resistant depression: A pilot randomised controlled trial |
| 25 | Sanjay Johan Mathew | 2008 | Optimization of IV Ketamine for Treatment Resistant Depression |
| 29 | NA | 2012 | A Study of Olanzapine and Fluoxetine for Treatment-resistant Depression |
| 30 | Corya, S. A. | 2006 | A randomized, double-blind comparison of olanzapine/fluoxetine combination, olanzapine, fluoxetine, and venlafaxine in treatment-resistant depression |
| 32 | Astra Zeneca | 2008 | Comparing Quetiapine XR Monotherapy and Augmentation With Lithium Augmentation in TRD Patients |
| 33 | Milena Antunes Santos | 2008 | Efficacy and safety of antidepressant augmentation with lamotrigine in patients with treatment-resistant depression: a randomized, placebo-controlled, double-blind study |
| 39 | McAllister-Williams | 2016 | Antidepressant augmentation with metyrapone for treatment-resistant depression (the ADD study): a double-blind, randomised, placebo-controlled trial |
| 41 | Palhano-Fontes | 2019 | Rapid antidepressant effects of the psychedelic ayahuasca in treatment-resistant depression: a randomized placebo-controlled trial |
| 43 | Yale University | 2010 | Efficacy and Tolerability of Riluzole in Treatment Resistant Depression |

**Table S3: Basic information of the included studies**

| **ID** | **Study** | **Treatment** | **Sample**  **size** | **Age**  **( year)** | **Female（%）** | **Baseline(score)** | **Dosing schedule** | **Frequency of Administration** | **Administration**  **Route** | **Dropout**  **rate, %** | **Duration (days)** |
| --- | --- | --- | --- | --- | --- | --- | --- | --- | --- | --- | --- |
| 01 | Vanina 2019 | Esketamine +SSRIs/SNRIs | 116 | 44.9 | 65.8 | 37 | 56 or 84mg for Esketamine | twice weekly for Esketamine added to a daily  SSRIs/SNRIs | IN+PO | 15.5 | 28 |
|  |  | SSRIs/SNRIs | 111 | 46.4 | 57.8 | 37.3 |  |  | PO | 10.8 | 28 |
| 02 | Maggie 2019 | Esketamine + SSRIs/SNRIs | 117 | 46.4 | 70.4 | 37.4 | 56mg for Esketamine | twice weekly for Esketamine added to a daily  SSRIs/SNRIs | IN+PO | 5.1 | 28 |
|  |  | Esketamine + SSRIs/SNRIs | 117 | 46.4 | 70.4 | 37.4 | 84mg for Esketamine |  | IN+PO | 5.1 | 28 |
|  |  | SSRIs/SNRIs | 113 | 46.8 | 71.7 | 37.5 | NA |  | PO | 5.3 | 28 |
| 04 | NCT03434041 | Esketamine + SSRIs/SNRIs | 126 | 36.8 | 46.0 | NA | 56 or 84 mg for Esketamine | twice weekly for Esketamine added to a daily  SSRIs/SNRIs | IN+PO | 14.3 | 28 |
|  |  | SSRIs/SNRIs | 126 | 37.8 | 43.7 | NA |  |  | PO | 15.9 | 28 |
| 05 | Ella 2018 | Esketamine +other Antidepressants | 11 | 42.1 | 45.5 | 31.3 | 28mg for Esketamine | twice weekly for Esketamine added to a daily oral antidepressant | IN+PO | 27.3 | 28 |
|  |  | Esketamine+ other Antidepressants | 11 | 42.7 | 81.8 | 33.2 | 56mg for Esketamine |  | IN+PO | 0 | 28 |
|  |  | Esketamine +other Antidepressants | 12 | 49.8 | 50.0 | 35 | 84mg for Esketamine |  | IN+PO | 16.7 | 28 |
|  |  | other Antidepressants | 33 | 44.9 | 54.5 | 35 | NA |  | PO | 0 | 28 |
| 06 | Jaskaran 2016 | Esketamine | 10 | 44.7 | 55.6 | 33.1 | 20 mg/kg for esketamine | twice weekly for Esketamine | IV | 0 | 7 |
|  |  | Esketamine | 11 | 41.8 | 63.6 | 33.7 | 40 mg/kg for esketamine |  | IV | 9.1 | 7 |
|  |  | Esketamine* | 10 | 42.7 | 60.0 | 30.8 | 20 or 40 mg/kg for esketamine |  | IV | 0 | 7 |
| 7 | Nagahide 2021** | Esketamine+ other Antidepressants | 41 | 45.9 | 56.1 | 38.4 | 28mg for Esketamine | twice weekly for Esketamine added to a daily oral antidepressant | IN+PO | 4.9 | 28 |
|  |  | Esketamine+ other Antidepressants | 40 | 42.5 | 40.0 | 37.9 | 56mg for Esketamine |  | IN+PO | 17.5 | 28 |
|  |  | Esketamine+ other Antidepressants | 41 | 41.9 | 43.9 | 35.9 | 84mg for Esketamine |  | IN+PO | 4.9 | 28 |
|  |  | other Antidepressants | 80 | 43.3 | 48.8 | 37.7 | NA |  | PO | 10.0 | 28 |
| 08 | Maurizio 2020** | Ketamine | 18 | 43.1 | 55.6 | 33.8 | 0.1mg/kg of Ketamine | twice for 3 days | IV | 22.2 | 3 |
|  |  | Ketamine | 20 | 45.5 | 45.0 | 34.5 | 0.2mg/kg of Ketamine |  | IV | 20.0 | 3 |
|  |  | Ketamine | 22 | 48.6 | 50.0 | 31.6 | 0.5mg/kg of Ketamine |  | IV | 4.5 | 3 |
|  |  | Ketamine | 20 | 47.4 | 40.0 | 32.7 | 1.0mg/kg of Ketamine |  | IV | 15.0 | 3 |
| 10 | Mu-Hong Chen 2019 | Ketamine | 16 | 43.3 | 68.8 | 33.3 | 0.2mg/kg of Ketamine | twice for 3 days | IV | NA | 3 |
|  |  | Ketamine | 16 | 44.4 | 68.8 | 32.1 | 0.5mg/kg of Ketamine |  | IV | NA | 3 |
| 12 | NCT03149991 | Ketamine+ Brexpiprazole | 25 | 40.8 | 56.0 | 33.8 | 3mg for Brexpiprazole and 40 mg for Ketamine | twice weekly for Ketamine added to a daily Brexpiprazole | IN+PO | 16.0 | 28 |
|  |  | Ketamine | 26 | 44.6 | 46.2 | 34.2 | 40 mg for Ketamine |  | IN+PO | 7.7 | 28 |
| 13 | NCT03756129 | MIJ821 | 21 | 51.15 | 34.1 | NA | 0.16 mg/kg | 11patients weekly;10patients biweekly | IV | 23.8 | 14 |
|  |  | MIJ821 | 19 | 44.75 | 63.3 | NA | 0.32 mg/kg | 10patients weekly;9patients biweekly | IV | 31.6 | 14 |
|  |  | Ketamine | 10 | 52.3 | 70.0 | NA | 0.5mg/kg | twice weekly | IV | 10.0 | 14 |
| 14 | Singh 2016 | Ketamine | 18 | 5.7 | 66.7 | 33.3 | 0.5mg/kg | 3 times weekly | IV | 33.3 | 15 |
|  |  | Ketamine | 17 | 43.3 | 70.6 | 35.4 | 0.5mg/kg |  | IV | 35.3 | 15 |
| 18 | NCT01880593 | Ketamine+ Lithium | 18 | 45 | 50.0 | 32.4 | 600-1200mg of Lithium daily night | twice weekly for Ketamine added to a daily Lithium | IV+PO | 44.4 | 14 |
|  |  | Ketamine | 16 | 45.87 | 56.3 | 32.8 | NA | NA | IV | 37.5 | 14 |
| 19 | Rebecca 2009 | Ketamine | 26 | 48.2 | 38.5 | 36.9 | 0.5mg/kg | twice weekly | IV | NA | 12 |
|  |  | Ketamine | 10 | 50.1 | 50.0 | 32.7 | 0.5mg/kg |  | IV | NA | 12 |
| 23 | Lenze 2016 | Ketamine+ clonidine | 10 | 46.6 | 80.0 | 34 | 0.6mg/kg | twice weekly | IV+PO | NA | 14 |
|  |  | Ketamine+ clonidine | 10 | 42.5 | 60.0 | 31.9 | 0.5mg/kg |  | IV+PO | NA | 14 |
| 25 | NCT00768430 | Ketamine | 48 | 46.9 | 56.3 | NA | 0.5mg/kg | once weekly | IV | 2.1 | 7 |
| 29 | NCT01687478 | Olanzapine + Fluoxetine | 88 | 38.63 | 46.6 | 32.1 | 5~15mg for Olanzapine 20~50mg for  Fluoxetine | once daily | PO | 36.4 | 56 |
|  |  | Fluoxetine | 88 | 41.45 | 40.9 | 31.8 | 20~50mg for Fluoxetine | once daily | PO | 44.3 | 56 |
| 30 | Corya 2006 | Olanzapine + Fluoxetine | 243 | 45.7 | 72.5 | 29.6 | olanzapine 13.5mg, fluoxetine 52mg | once daily | PO | 24.7 | 84 |
|  |  | Olanzapine | 62 | 45.7 | 72.5 | 29.6 | 12.5mg |  | PO | 29.0 | 84 |
|  |  | Fluoxetine | 60 | 45.7 | 72.5 | 29.6 | fluoxetine 52mg |  | PO | 20.0 | 84 |
|  |  | venlafaxine | 59 | 45.7 | 72.5 | 29.6 | 75~375mg |  | PO | 25.4 | 84 |
|  |  | Olanzapine + Fluoxetine | 59 | 45.7 | 72.5 | 29.6 | olanzapine6.5mg fluoxetine 25mg |  | PO | 22.0 | 84 |
| 32 | NCT00789854 | Quetiapine | 225 | 47 | 67.6 | 33.74 | 300mg | once daily | PO | 21.5 | 42 |
|  |  | Quetiapine +SSRIs/SNRIs | 229 | 47 | 70.7 | 33.15 | 300mg for Quetiapine |  | PO | 15.2 | 42 |
|  |  | Lithium +SSRIs/SNRIs | 221 | 47 | 68.8 | 32.91 | 900mg  for Lithium |  | PO | 20.5 | 42 |
| 33 | Milena 2008 | Lamotrigine +other Antidepressants | 17 | 26 | 82.4 | 32.3 | 50~200mg for Lamotrigine | once daily | PO | 17.6 | 56 |
|  |  | other Antidepressants | 17 | 29 | 64.7 | 28.4 | NA |  | PO | 23.5 | 56 |
| 39 | McAllister 2016 | Metyrapone +other Antidepressants | 83 | 47.6 | 56.6 | 27.7 | NA | once daily | PO | 16.9 | 35 |
|  |  | Antidepressant | 82 | 47.6 | 56.6 | 27.7 | NA |  | PO | 9.8 | 35 |
| 41 | Palhano 2019 | Ayahuasca | 17 | 39.71 | 78.6 | 36.1 | 0.36mg/kg | once weekly | IV | 17.6 | 7 |
| 43 | NCT01204918 | Riluzole + SSRIs/SNRIs | 25 | 46.3 | 36.0 | NA | 100mg for Riluzole | once daily | PO | 16.0 | 56 |
|  |  | Riluzole + SSRIs/SNRIs | 39 | 47.3 | 61.5 | NA | 100mg for Riluzole |  | PO | 10.3 | 56 |
|  |  | SSRIs/SNRIs | 40 | 46.3 | 52.5 | NA | NA |  | PO | 12.5 | 56 |

NA: not report. PO represents oral administration, IV represents intravenous administration, and IN represents intranasal administration.

*The group received placebo for the first dose, and Esketamine was administered starting with the second dose

**The trial was not blinded after the third day of injection

**Table S4. Risk assessment for each included study**

| **Study** | **Randomization process** | **Deviations from intended interventions** | **Missing outcome data** | **Measurement of the outcome** | **Selection of the reported result** | **Overall Bias** |
| --- | --- | --- | --- | --- | --- | --- |
| Vanina 2019 | Low | Low | Low | Low | Low | Low |
| Maggie 2019 | Low | unclear | Low | Low | Low | unclear |
| NCT03434041 | Low | unclear | Low | Low | Low | unclear |
| Ella 2018 | Low | unclear | Low | Low | unclear | unclear |
| Jaskaran 2016 | Low | Low | Low | Low | Low | Low |
| Nagahide 2021 | Low | Low | Low | Low | Low | Low |
| Maurizio 2020 | Low | Low | Low | Low | unclear | unclear |
| Mu-Hong Chen 2019 | Low | unclear | unclear | Low | unclear | unclear |
| NCT03149991 | Low | Low | Low | Low | Low | Low |
| NCT03756129 | Low | unclear | Low | Low | unclear | unclear |
| Singh 2016 | Low | Low | Low | Low | Low | Low |
| NCT01880593 | Low | Low | Low | Low | unclear | unclear |
| Rebecca 2009 | Low | Low | Low | Low | High | High |
| Lenze 2016 | Low | Low | Low | Low | unclear | unclear |
| NCT00768430 | Low | Low | Low | Low | High | High |
| NCT01687478 | Low | Low | Low | Low | Low | Low |
| Corya 2006 | Low | Low | unclear | Low | Low | unclear |
| NCT00789854 | High | Low | Low | Low | unclear | High |
| Milena 2008 | Low | Low | Low | Low | unclear | unclear |
| McAllister 2016 | Low | Low | Low | Low | Low | Low |
| Palhano 2019 | Low | Low | Low | Low | Low | Low |
| NCT01204918 | Low | Low | Low | Low | unclear | unclear |

**Figure S3. The summary risk of literature assessment**

**
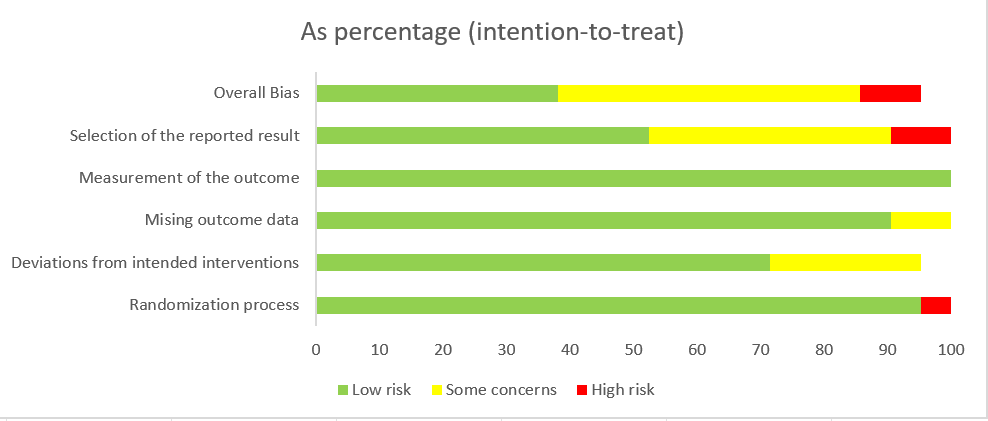
**

**Covariate analysis**

**Figure S4. Correlation analysis between model parameters and covariates in the oral administration model**


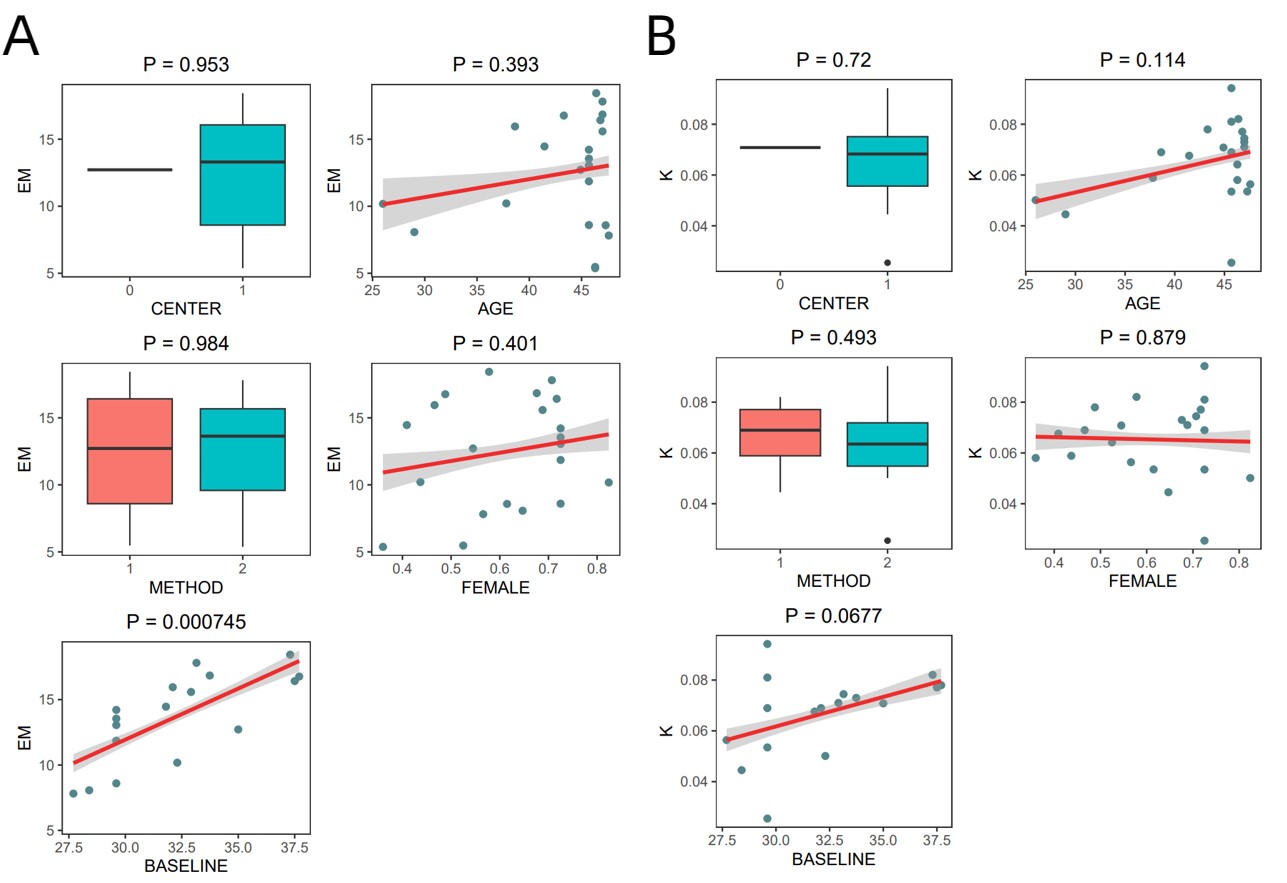


The correlation between continuous variables and the model parameters E_max_ (Panel A) and K (Panel B) was assessed using Pearson correlation analysis. The association between categorical variables and model parameters was evaluated using the t-test. A p-value of less than 0.05 was considered significant, indicating a statistically significant correlation.

EM: E_max_; CENTER=0: Non-international multicenter clinical trial, CENTER=1: International multicenter clinical trial; AGE: Age of subjects; METHOD=1: monotherapy administration, METHOD=2: Combined administration; FEMALE: Proportion of female subjects; BASELINE: Baseline of Montgomery-Åsberg Depression Rating Scale (MADRS) score.

**Figure S5. Correlation analysis between model parameters and covariates in the intravenous administration model**


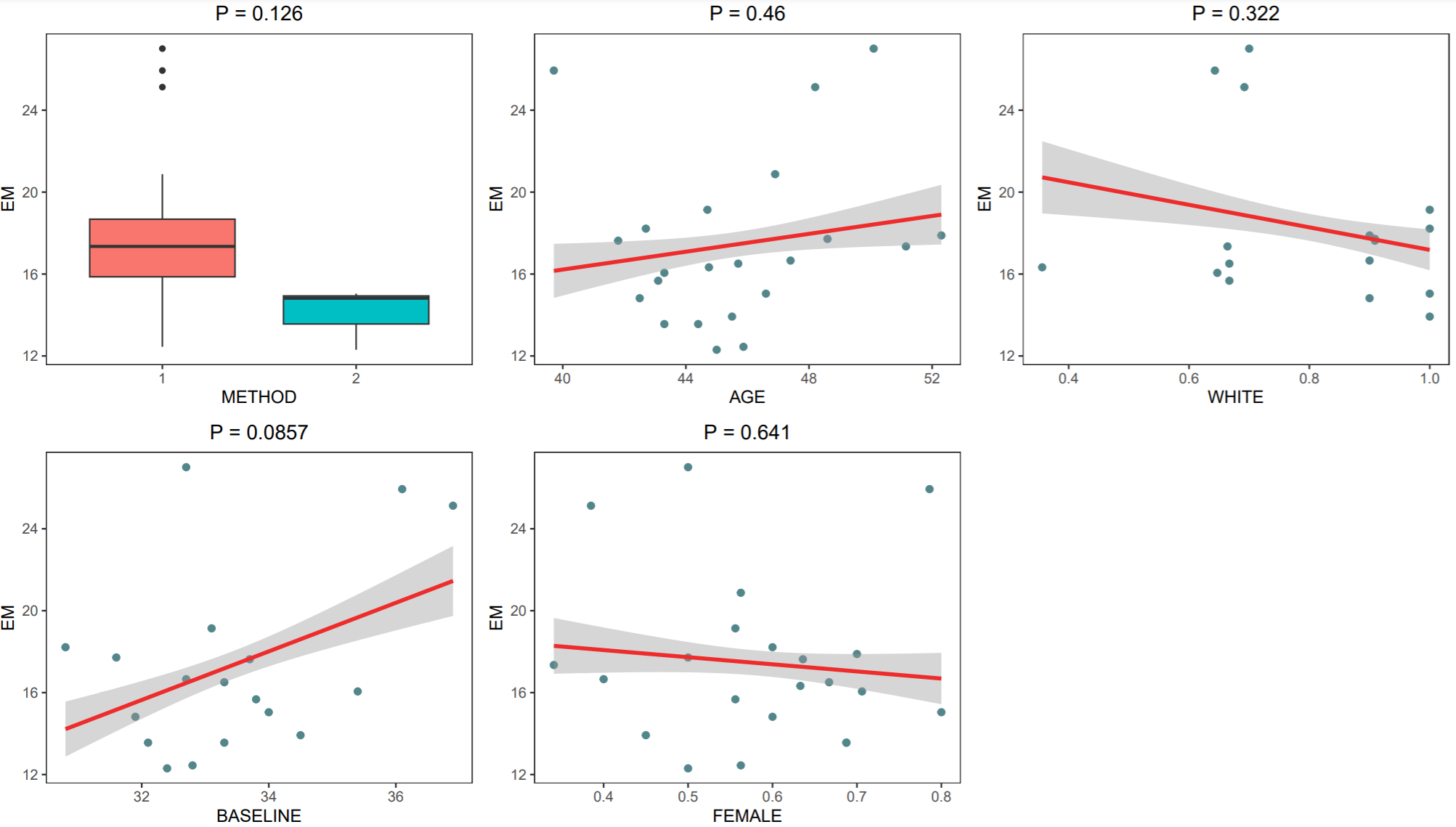


The correlation between continuous variables and the model parameters E_max_ was assessed using Pearson correlation analysis. The association between categorical variables and model parameters was evaluated using the t-test. A p-value of less than 0.05 was considered significant, indicating a statistically significant correlation.

EM: E_max_; METHOD=1: monotherapy administration, METHOD=2: Combined administration; AGE: Age of subjects; WHITE: Proportion of White subjects; BASELINE: Baseline of Montgomery-Åsberg Depression Rating Scale (MADRS) score; FEMALE: Proportion of female subjects.

**Figure S6. Correlation analysis between model parameters and covariates in the intranasal administration model**


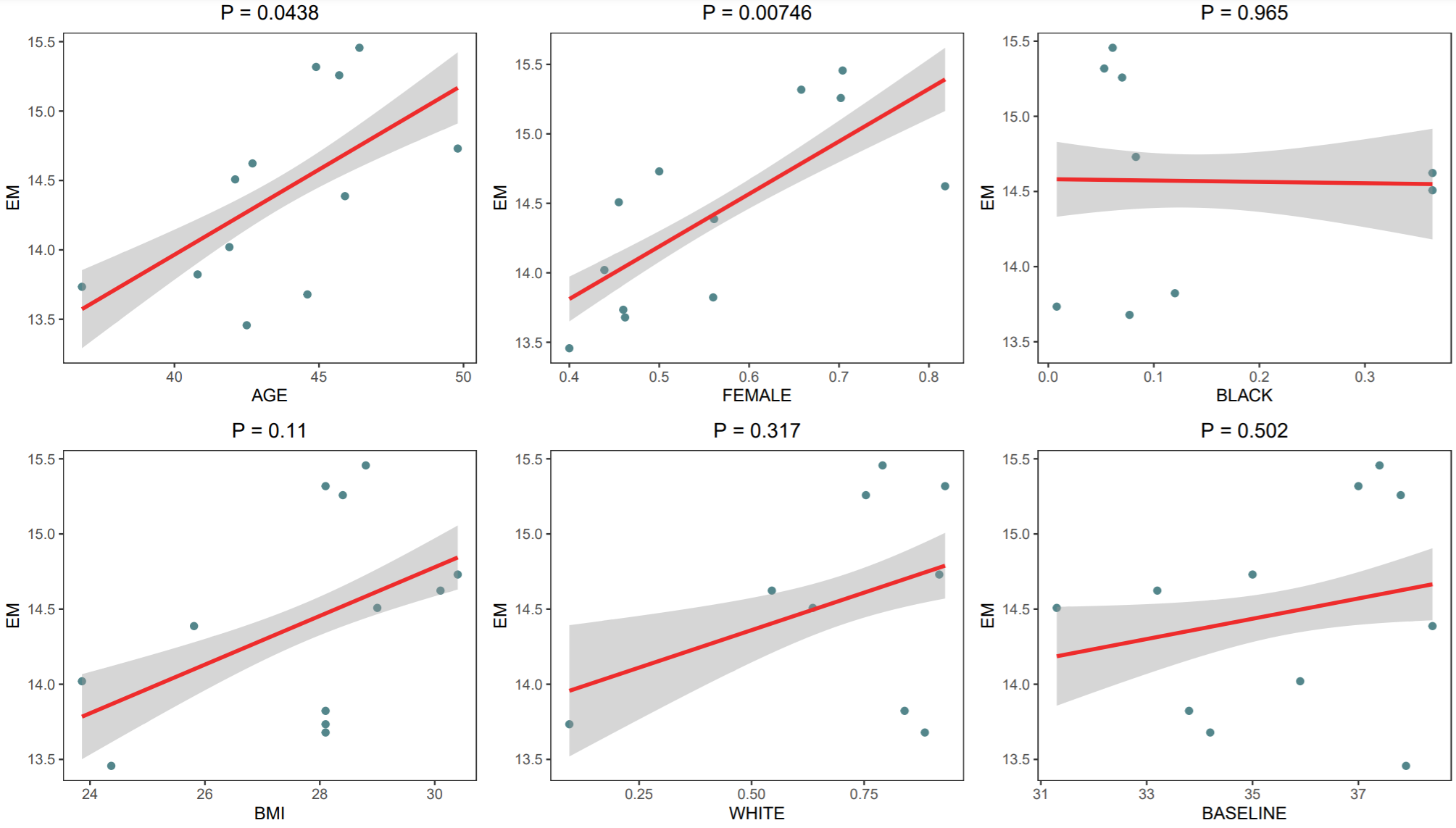


The correlation between continuous variables and the model parameters Emax was assessed using Pearson correlation analysis. The association between categorical variables and model parameters was evaluated using the t-test. A p-value of less than 0.05 was considered significant, indicating a statistically significant correlation.

EM: E_max_; AGE: Age of subjects; FEMALE: Proportion of female subjects; BLACK: Proportion of Black subjects; WHITE: Proportion of White subjects; BASELINE: Baseline of Montgomery-Åsberg Depression Rating Scale (MADRS) score.

**Figure S7. The goodness-of-fit plots of oral administration model**

**
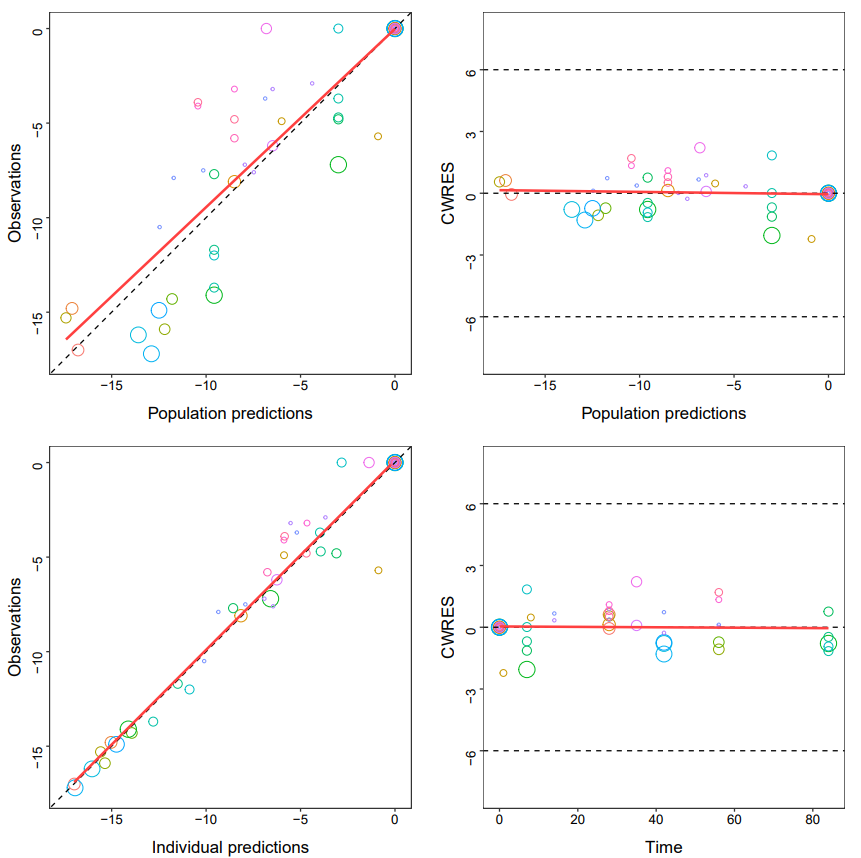
**

The top-left graph displays the population predictions versus the observed values, while the bottom-left graph illustrates the individual predictions against observed values. The top-right graph depicts the conditional weighted residuals (CWRES) versus population predictions, and the bottom-right graph presents CWRES against time. In the top-left and bottom-left graphs, dashed lines represent the diagonal and solid lines indicate the fitting lines. Similarly, in the top-right and bottom-right graphs, dashed lines denote the 0 and ±6 lines, and solid lines represent the fitting lines. The color coding of the points corresponds to different trial groups.

**Figure S8. The goodness-of-fit plots of intravenous administration model**

**
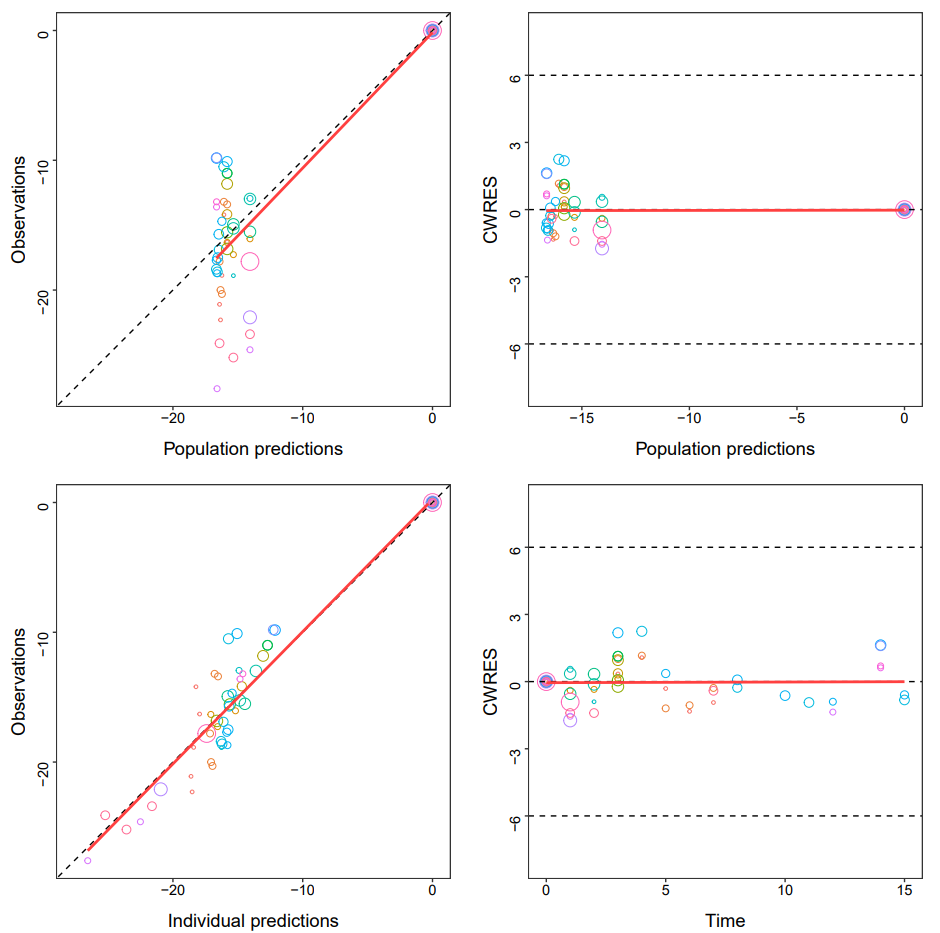
**

The top-left graph displays the population predictions versus the observed values, while the bottom-left graph illustrates the individual predictions against observed values. The top-right graph depicts the conditional weighted residuals (CWRES) versus population predictions, and the bottom-right graph presents CWRES against time. In the top-left and bottom-left graphs, dashed lines represent the diagonal and solid lines indicate the fitting lines. Similarly, in the top-right and bottom-right graphs, dashed lines denote the 0 and ±6 lines, and solid lines represent the fitting lines. The color coding of the points corresponds to different trial groups.

**Figure S9. The goodness-of-fit plots of intranasal administration model**

**
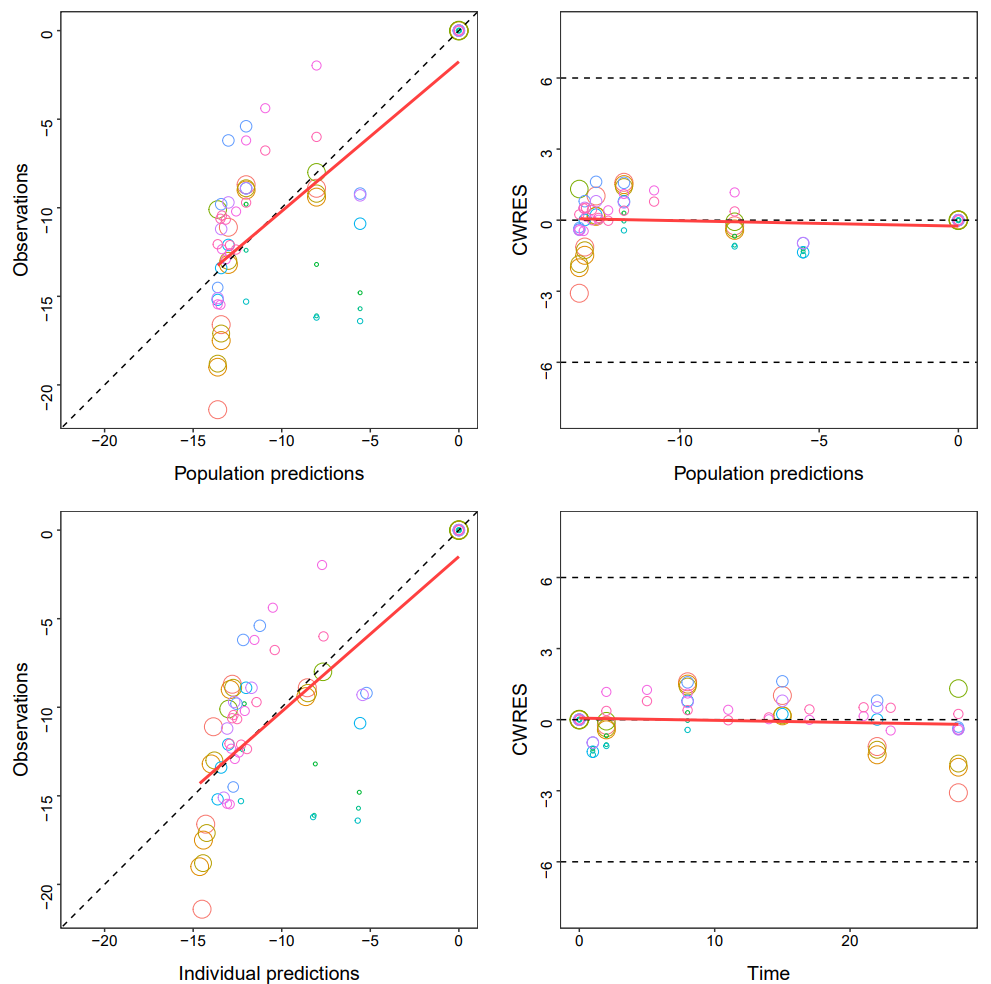
**

The top-left graph displays the population predictions versus the observed values, while the bottom-left graph illustrates the individual predictions against observed values. The top-right graph depicts the conditional weighted residuals (CWRES) versus population predictions, and the bottom-right graph presents CWRES against time. In the top-left and bottom-left graphs, dashed lines represent the diagonal and solid lines indicate the fitting lines. Similarly, in the top-right and bottom-right graphs, dashed lines denote the 0 and ±6 lines, and solid lines represent the fitting lines. The color coding of the points corresponds to different trial groups.
